# Morbidity and Mortality Conference in Emergency Medicine Residencies and the Culture of Safety

**DOI:** 10.5811/westjem.2015.8.26559

**Published:** 2015-10-22

**Authors:** Emily L. Aaronson, Kathleen A. Wittels, Eric S. Nadel, Jeremiah D. Schuur

**Affiliations:** * Brigham and Women’s Hospital, Department of Emergency Medicine, Boston, Massachusetts; † Massachusetts General Hospital, Department of Emergency Medicine, Boston, Massachusetts; ‡ Harvard Medical School, Department of Emergency Medicine, Boston, Massachusetts

## Abstract

**Introduction:**

Morbidity and mortality conferences (M+M) are a traditional part of residency training and mandated by the Accreditation Counsel of Graduate Medical Education. This study’s objective was to determine the goals, structure, and the prevalence of practices that foster strong safety cultures in the M+Ms of U.S. emergency medicine (EM) residency programs.

**Methods:**

The authors conducted a national survey of U.S. EM residency program directors. The survey instrument evaluated five domains of M+M (Organization and Infrastructure; Case Finding; Case Selection; Presentation; and Follow up) based on the validated Agency for Healthcare Research & Quality Safety Culture survey.

**Results:**

There was an 80% (151/188) response rate. The primary objectives of M+M were discussing adverse outcomes (53/151, 35%), identifying systems errors (47/151, 31%) and identifying cognitive errors (26/151, 17%). Fifty-six percent (84/151) of institutions have anonymous case submission, with 10% (15/151) maintaining complete anonymity during the presentation and 21% (31/151) maintaining partial anonymity. Forty-seven percent (71/151) of programs report a formal process to follow up on systems issues identified at M+M. Forty-four percent (67/151) of programs report regular debriefing with residents who have had their cases presented.

**Conclusion:**

The structure and goals of M+Ms in EM residencies vary widely**.** Many programs lack features of M+M that promote a non-punitive response to error, such as anonymity. Other programs lack features that support strong safety cultures, such as following up on systems issues or reporting back to residents on improvements. Further research is warranted to determine if M+M structure is related to patient safety culture in residency programs.

## INTRODUCTION

### Background

Following the Institute of Medicine’s (IOM) 1999 report To Err is Human,^1^ there has been widespread support for promoting a culture of safety within healthcare organizations.^2^ A key goal of the patient safety movement has been creating non-punitive systems that encourage approaching safety systematically.^3^ Routine case reporting and detailed case review is an essential art of this systematic approach, and facilitates the evaluation of clinical judgment and identification of systems errors. If conducted with attention to best practices such as non-punitive review, debriefing, and follow up on systems improvements it can support building strong safety cultures in medicine.^4^

### Importance

Morbidity and mortality conferences (M+Ms) hold a long tradition in medicine and play important roles in physician education and quality improvement (QI).^5^ However, the historical culture of “blame and shame” embedded in M+M conferences is at odds with the goals of educating trainees in a culture of safety.^6^,^7^ M+M conferences structured to teach residents to systematically analyze practice using QI methods in a non-punitive environment can help enhance emergency medicine (EM) safety culture.^8^ Specifically, recommended techniques include conference formats that employ anonymous case reporting, use non-punitive approaches to case review, formal debriefing of trainees with cases, and follow up of actions taken to address systems issues.^9^–^11^ While there was one published survey studying the formats of M+M in EM residency programs,^12^ this did not focus on the methods that M+M uses to approach non-punitive review, debriefing, and follow up.

### Goals of this investigation

This study aimed to determine the structure and processes of M+Ms in U.S. EM residency programs. Specifically, we were interested in determining the proportion of EM residency programs with conferences that were structured to 1) provide anonymous case submission and maintain anonymity during case presentations, 2) provide debriefing with residents after conferences are complete, and 3) follow up on systems issues identified during M+M.

## METHODS

### Study Design

We conducted a cross-sectional survey of all U.S. EM residency programs in the Society for Academic Emergency Medicine (SAEM) directory to determine the structure of their M+M conferences. We designed a 19-item survey instrument to assess the structure of EM M+M conferences in the context of safety culture. The survey instrument evaluated five domains of M+M conferences: 1) organization and infrastructure; 2) case finding; 3) case selection; 4) presentation; and 5) follow up. The domains and culture of safety questions were based on the previously validated Agency for Healthcare Research & Quality (AHRQ) Patient Safety Survey.^13^ The survey was pilot-tested on three chief residents for clarity prior to survey distribution. The survey was administered using a web-based survey tool (Survey Monkey, Palo Alto, CA) and is attached as an appendix. If a respondent did not answer all questions, their complete answers were included. Our institutional review board approved this study.

### Study Setting and Population

We surveyed every EM residency listed in the SAEM directory between September and December 2013. The survey was to be completed by the individual most responsible for overseeing M+M conferences at their institution. We emailed the survey to the program director, who was instructed to complete the survey or forward it on to the individual most responsible for M+M. Non-responders received repeat email requests and follow-up phone calls to encourage completion.

### Key Outcome Measures

We defined anonymity as complete when neither residents nor attending physicians involved in the case were named in the case presentation, responsible for presenting it, or asked to comment during the case presentation. We defined anonymity as partial when neither residents nor attending physicians involved in the case were named in the case presentation or were responsible for presenting it, but could be asked to comment on it. Other outcomes including debriefing and follow up on systems improvement reflect the survey questions (See Appendix).

### Data Analysis

We calculated descriptive statistics and 95% confidence intervals (95% CI). Bivariate associations were assessed with the Chi-square test. P<0.05 was considered significant.

## RESULTS

### Characteristics of study subjects

In the spring of 2013, 188 active EM training programs were listed in the SAEM directory. We received 164 unique responses, but had to exclude 13 as the survey response did not provide identifying information for the specific program. We include 151 responses from specific residency programs (80% response rate). One hundred forty-six out of the 151 (97%) respondents answered all questions. The demographics of responding programs can be found in Table 1. The majority of surveys were completed by residency physician administrators (108/151, 72%), with the remaining surveys completed by ED quality or safety leaders (17/151, 11%), ED clinical directors or operations administrators (11/151, 7%), attendings with no formal title (8/151, 5%) or the ED chairs (7/151, 5%). All respondents were attending physicians.

### Structure

The basic characteristics of M+M conferences are reported in Table 2. When respondents were asked to rank, in order of importance, the objectives of M+M, the most common primary objective was to discuss adverse outcomes (53/151, 35%), followed by identify systems errors (47/151, 31%), identify cognitive errors (26/151, 17%), discuss interesting cases (15/151, 10%), teach individual professional accountability (6/151, 4%), and “other” (4/151, 3%).

### Case finding and selection

Cases for M+M conferences were identified by multiple methods (Table 2), with email from providers (124/146, 85%) and hospital patient safety reporting system (115/146, 79%) used most frequently. Regular review of ED deaths and return visits were done at the majority of institutions (93/146, 64% and 81/146, 55% respectively), while regular review of death after admission was performed at 41% of institutions.

The decision regarding which cases to include in the conference was made by the attending supervising the conference at 40% (61/151) of institutions and through collaboration between the resident presenting and the attending supervising the conference at 40% (60/151) of institutions. At the remaining institutions, the resident presenting the cases (10/151, 7%), QI leadership (8/151, 5%) and chief residents (6/151, 4%) decided which cases to present. Six institutions (4%) listed other mechanisms for choosing which cases to present, such as an education fellow or QI committee.

When asked to rank in order of importance, the criteria used to determine which cases were presented, the most frequently top-rated criteria was the presence of errors, regardless of patient outcome (73/150, 49%), followed by severity of outcome (42/150, 28%), interesting nature of disease (29/150, 19%) and referral by another department (6/150, 4%).

### Case presentation and anonymity

**Figure 1:**
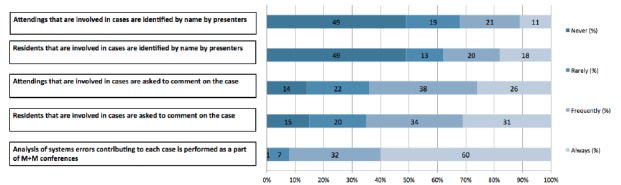
Structure of morbidity and mortality conferences (M+M) case presentation.

The structure of case presentation varies across residencies (Table 2). There is variation related to anonymity, both in case submission and during the conference itself (Figure 1). Ten percent (15/151) of programs maintained complete anonymity during the case presentation, 21% (31/151) of program maintained partial anonymity, and 69% (105/151) did not maintain anonymity. We were unable to detect any difference between the proportion of programs with complete and partial anonymity across programs of different size, location or the length of residency training (three years vs. four).

### Follow up

Forty-seven percent (71/151) of programs have a formalized process for following up on systems issues identified at M+Ms. This was not different across programs of different size, location or the length of residency training. The changes made as a result of cases presented at M+M conferences are reported back at future M+Ms at 10% (15/151) of programs, and by email or another method at 58% (88/151) of programs. The remaining 32% (48/151) of programs do not regularly report back on changes made.

Forty-four percent (67/151) of programs report that they regularly debrief with residents who have had cases discussed. When this is done, it is most often done by a member of the residency administration (39/151, 26%) and less often by a chief resident (3/151, 2%) or someone else (25/151, 17%). The proportion of programs with a formalized process in place for following up on systems issues was not different across programs of different size, location or the length of residency training. There was also no difference between these variables and the proportion of programs that have a regular debriefing for residents who have had their cases presented.

There is a system to evaluate M+M conferences in place at most institutions, with attending physicians evaluating conferences at 61% (92/151) of institutions, and residents at 66% (100/151). Fifty-two percent (79/151) of institutions report that both resident and attending physicians formally evaluate these conferences.

**Figure 2:**
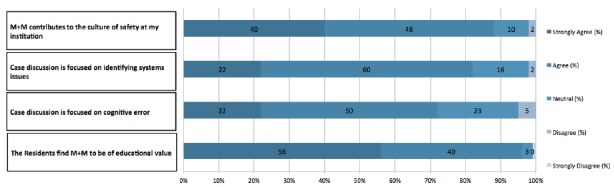
Goals and impact of morbidity and mortality conferences (M+M) conference.

The majority of respondents believe that M+M conferences are of educational value to the residents (144/150, 96%) (Figure 2). Most respondents also believe that case discussion focuses on identifying systems errors (124/151, 82%) and identifying cognitive errors (109/151, 72%). Eighty-eight percent (133/151) of respondents believe that M+M contributes to the culture of safety at their institutions.

## DISCUSSION

M+M conferences, a requirement of the Accreditation Counsel of Graduate Medical Education (ACGME) Resident Review Committee, serve a key quality and safety function for departments of EM across the U.S. We surveyed U.S. EM residencies and found variability in the organization and structure of these conferences. Although best practice suggests that high quality incident analysis requires robust reporting, non-punitive review, and institutionalized follow up and debriefing,^4^ we found that many EM programs have not implemented these best practices in their M+M conferences.

The concept of safety cultures, born out of error analysis in Chernobyl,^14^,^15^ has been adapted from other high-reliability industries and widely applied in healthcare. The IOM has further reinforced the essential role that physicians play in creating a strong safety culture through voluntary reporting of error.^1^

EM residency programs do not appear to have standardized this process. Of note, one fifth of programs do not use a hospital patient safety reporting system to identify cases, and EM programs are as likely to use email submissions as they are to use their hospital’s patient safety reporting systems. The risk of this practice is that it bypasses institutional safety analysis, and may leave out certain stakeholders such as nursing or other relevant departments.

This lack of structured voluntary reporting is not surprising. EM also lacks an industry-wide standard for which incidents mandate peer review, likely contributing to the variation that we found in the criteria used to determine which cases are reviewed. While the Joint Commission and state boards have standards for mandatory reporting, such as perioperative death and wrong-side surgery, there are not similar standards or guidelines of which cases EDs should be reviewing.

We did, however, find that most programs are reviewing a common set of indicators including ED deaths and return visits. However, about a fifth are not reviewing hospital patient safety reports, over a third of programs are not routinely reviewing ED deaths, and even less are reviewing deaths during the inpatient stay. As deaths are the highest-risk cases, this raises concern that systematic review of all errors is not done at many EM programs. Inpatient mortality is also increasingly important to hospitals as Medicare expands the measurement of 30-day hospital mortality and increases the amount of reimbursement that is tied to performance on this metric. Given this, inpatient deaths soon after ED admission (e.g. 48 hours) should be included in standard EM case reviews.

Anonymity during incident reporting is one technique to encourage robust reporting and a strong safety culture.^16^,^17^ We found that anonymity during M+M is not the norm at many EM programs. Just over half of programs provide anonymous case reporting, and only 10% structure their conferences to keep both attending physicians and residents completely anonymous during case discussion. Extrapolating from the evidence supporting anonymous reporting, one could posit that anonymous case review would further reinforce a culture of safety. Given that trainees are a vulnerable population, we suspect that they may experience public review of their role in adverse events as humiliating, shameful and ultimately punitive. Indeed, a survey of trainees by Wu et al. found that trainees who publicly accept responsibility for error undergo significant emotional stress, and that these events are associated with remorse, anger, guilt, and feelings of inadequacy.^18^ Interestingly, in this survey, it was also noted that residents who publicly discussed their cases were more likely to report constructive changes in their practice.

This paradox–the perception that punitive environments can foster learning–is one that has likely prevented more widespread adoption of anonymous M+M conferences. Despite the potential educational effect of a punitive behavior, the cost associated with the emotional stress and disincentive to report has lead to an industry-wide movement towards non-punitive healthcare environments. This movement represents a paradigm shift from the historical structure of case review at M+M, which focused on holding individuals personally accountable for errors, regardless of contributing factors. This “blame and shame” approach hinged on identifying an individual responsible for an adverse event and encouraging critical comments from peers and superiors on the nature of the error. However, with only 4% of programs ranking “teaching personal accountability” as their primary objective of M+M, it seems that this cultural shift has begun. We posit that although programs’ objectives are aligned with non-punitive review, they have been slower to make their M+M process anonymous because of these historical traditions. Indeed, with only 44% of hospitals surveyed in the most recent AHRQ Hospital Survey on Patient Safety Culture endorsing non-punitive response to error, this is a persistent problem nationally.^19^

There are likely several reasons that M+M has been slow to adopt a non-punitive approach to case review. The individuals in charge of M+M, generally senior physicians with administrative roles, trained in the era of “blame and shame” M+M. While they may understand the importance of changing M+M culture to support non-punitive review, they may not appreciate the stress and negative feelings that having one’s case publicly presented at M+M causes for the average provider, especially for trainees. Second, we suspect that some physicians believe that anonymity reduces personal accountability and do not recognize the role that it plays in creating a safe space which encourages self-reporting of cases and maximizes learning.

Indeed, even within our authorship group there is not complete agreement on the role that anonymous case review plays in creating non-punitive cultures. While three of us think an anonymous M+M is more supportive of safety culture (EA, KW, JS), one of us (EN) thinks that naming providers in M+M can improve professional development and contribute to a culture of safety if done in a supportive environment. However, we all agree that anonymity may allow for more effective engagement of audience members, allowing them to separate the error from the individuals being discussed making these errors more teachable moments. Rather than the audience focusing on why that physician made the choices s/he did, they can instead focus on the generalizable systems and cognitive issues that apply to all providers presented with a similar patient, improving the educational benefit of the discussion. Given that the majority of institutions reported that they aim for the conferences to address systems issues, fostering anonymity should support this goal. Interestingly, to our knowledge, the effect of anonymity in medical case review on creating a non-punitive culture has not been researched.

Formal debriefing with residency leadership is important to assess the resident’s reaction to the adverse event, self-assessment, and development of a performance improvement plan if needed. If M+M presentations are anonymous, such debriefing can insure that trainees consider personal accountability for adverse events, an important characteristic of a professional culture. Less than half of programs, however, have regular debriefing with residents who have had their cases discussed at M+M. This represents another opportunity to use cases for physician education.

Research has shown that even highly capable individuals are prone to failure if working within a poorly designed system.^20^ Understanding this, and the multi-factorial nature of errors, it is essential that there are mechanisms in place to follow up on the systems issues identified at M+M conferences. Despite this, we found that only half of programs have a single individual who is responsible for following up on systems issues that are identified and even fewer have clear processes in place to do this. When changes are made, only a third of programs regularly report them back to staff. This may be because M+M conferences fall within the ED’s educational domain rather than operations, and could be improved if M+M was integrated as a component of quality, safety and operations effort. Indeed, research has shown that by implementing a hospital-wide M+M conference with the express purpose of focusing on systems-based problems hospitals can effectively engage multiple stakeholders in the open discussion of error, identification of system failures and promotion of initiatives to improve patient safety.^21^

The lack of formalized follow up may be why only a small minority (11%) of respondents believed that systems issues identified at M+M conferences always lead to change in their EDs. It has been well documented that a leading barrier to a culture of safety is a failure to follow up with frontline providers on how adverse events led to systems improvements.^22^,^23^ Without such follow up, frontline providers can feel that their observations about safety issues are not important, and if reported will fall on deaf ears, ultimately leading to a lower likelihood of reporting safety issues. This can deprive an ED of its most important source of information, as near misses and potential errors are much more common than adverse events that leaders usually hear about.

While we found that most EM M+Ms are not structured in a way that maximally supports strong safety cultures, we did not evaluate participants’ perceptions of M+M or safety culture. Further study is needed to evaluate the impact of M+M structure on residents’ perceptions of safety culture in their institutions and whether different M+M formats, such as those using anonymity, result in a less punitive culture and ultimately improved patient outcomes.

From review of this data and the literature on M+M, we recommend that programs prioritize the implementation of anonymous case reporting, creating a formalized process for follow up on the systems issues discussed and employing a clear structure for debriefing residents whose cases were discussed. Institutions can start this process by creating resident anonymity–recognizing that residents are a more vulnerable population for whom addition steps should be taken to ensure a non-punitive environment–while attendings continue to be publicly accountable for their cases. Together, these relatively basic changes will help promote robust case reporting and disclosure of error, helping our specialty set the example for strong safety cultures in residency training and incident analysis.

## LIMITATIONS

Because this was a survey, we were limited by response bias; the results represent the views of the individual respondent at those programs that responded. Our high response rate supports the external generalizability of our findings. All responses are self-reported and cannot be confirmed, another limitation of the study design. Additionally, only program directors or faculty directly responsible for M+Ms were surveyed. This results in a bias towards the opinions of those most responsible for conducting these conferences and does not represent the opinions of residents or other non-invested attending physicians. It is likely that the opinions program directors and faculty directly responsible for M+M are more positive than a general M+M audience, for example their assessment of the role of M+M on culture of safety, but also the presence of structures that would be perceived as positive, such as resident debriefing. Therefore, the proportion of residencies without structures that are perceived as positive is likely a conservative estimate. While the survey was anonymous–we did not collect individual identifiers–we did collect role and institution. Although survey respondents knew that the results would be reported anonymously, the survey was not anonymous to the study staff. This could have lead to misreporting.

As a survey, the questions asked also may not capture the nuances of many M+M conferences. For example, at some institutions cases may be presented anonymously; however, the residents and attendings involved may regularly volunteer remarks about their thought processes. These institutions would be classified as anonymous in this survey, which does not reflect the culture of safety that embraces people sharing details of error publicly, even if not asked.

## CONCLUSION

This national survey of EM residencies demonstrates that while M+M conference is a standard part of EM residency training in the U.S, there is a great degree of variation in the structure of these conferences. Many programs have not integrated key tenets of a culture of safety into their M+M process, such as anonymous case review, debriefing of participants and follow up of changes that resulted from the review. While this survey could not determine the impact of M+M structure on resident education and clinical practice, it demonstrates the opportunity for EM to improve the culture of safety by incorporating these elements into regular case review in the future.

## Supplementary Information



## Figures and Tables

**Table 1:** Demographics of responding emergency medicine residency programs.

	N	% (95% CIs)
Region		
Northeast	47	31 (18–44)
Midwest	41	27 (13–41)
South	41	27 (13–41)
West	22	15 (0.08–30)
Program structure		
3 Year	103	68 (59–77)
4 Year	48	32 (19–45)
Program size (total number of residents)		
0–20	17	11 (0–26)
21–40	83	55 (42–68)
41–60	48	32 (16–48)
>60	3	2 (0–75)

**Table 2:** Characteristics of emergency medicine morbidity and mortality conferences.

	n	% (95% CIs)
Organization and infrastructure		
Conference frequency		
Weekly	17	11 (0–26)
Bi-weekly (every other week)	10	7 (0–23)
Monthly	108	72 (64–81)
Less than once monthly	16	10 (0–25)
Conference length		
Shorter than 1 hour	3	2 (0–18)
1 hour	115	76 (68–84)
2 hours	27	18 (4–33)
Longer than 2 hours	6	4 (0–20)
Case finding		
Method for case identification		
Email from providers	124	85 (79–91)
Hospitals patient safety reporting system	115	79 (72–86)
Referred from risk management	98	67 (58–76)
Regular review of deaths in ED	93	64 (54–74)
Regular review of deaths after admission	60	41 (27–53)
Regular review of return visits	81	55 (44–66)
Anonymous case submission available		
Yes	84	56 (45–67)
No	67	44 (32–56)
Case selection		
Conference oversight		
Program director	37	25 (11–39)
Associate/assistant program director	22	15 (1–30)
Director of quality	54	36 (23–49)
Other faculty	35	24 (10–38)
Criteria used to determine which cases are presented		
Presence of errors, regardless of patient outcome	73	49 (41–57)
Severity of outcome	42	28 (21–35)
Interesting nature of disease	29	19 (13–25)
Referred by another department for presentation	6	4 (1–7)
Presentation		
Case presenter		
Resident involved in patient’s care	60	41 (29–53)
Resident who presents entire conference (not involved in patient’s care)	61	42 (30–54)
Faculty involved in patient’s care	7	5 (0–21)
Faculty not involved	18	12 (0–28)
Anonymity		
Maintain complete anonymity	15	10 (0–25)
Maintain partial anonymity	31	21 (7–35)
Follow up		
Single individual responsible for follow up		
Yes	76	50 (39–61)
No	75	50 (39–61)
Is there a formalized process for following up on systems issues identified at M+M		
Yes	71	47 (35–57)
No	79	53 (42–64)
Changes are made as a result of cases presented at M+M conferences reported back to residents		
Yes	103	68 (59–77)
Not regularly	48	32 (19–45)
There is regular debriefing with residents who have had their cases discussed at M+M		
Yes	67	44 (32–59)
No	84	56 (45–67)
M+M is formally evaluated by attendings		
Yes	92	61 (51–71)
No	59	39 (27–51)
M+M is formally evaluated by residents		
Yes	100	66 (57–75)
No	51	34 (21–47)

*ED*, emergency department

*M+M*, morbidity and mortality conferences
