# Supplementary material for: Morbidity and Mortality Conference in Emergency Medicine Residencies and the Culture of Safety
Source: West J Emerg Med. 2015 Oct 22;16(6):810–7. doi: 10.5811/westjem.2015.8.26559 (PMC4651575; doi:10.5811/westjem.2015.8.26559)
Supplement: Supplementary file 1 [file wjem-16-810-s001.pdf]

ORGANIZAITON AND INFRASTRUCTURE

1. What is your primary role in the Emergency Department?

- ◯ Residency Administration
- ◯ ED Quality or Safety Administration
- ◯ ED Director or Operations Administration
- ◯ Department Chief
- ◯ Attending with no formal Administrative title

2. How often does your residency program hold M&M conference?*
*If multiple sites, please include all clinical sites when determining frequency

- ◯ Weekly
- ◯ Bi-weekly (every other week)
- ◯ Monthly
- ◯ Less than once monthly

3. How long is M&M conference?

- ◯ Shorter than1 hour
- ◯ 1 hour
- ◯ 2 hours
- ◯ Longer than 2 hours

4. What is the objective of M&M?
Drag and drop the following choices in order of preference, with your top choice in the first position.

- [ ] Discuss adverse outcomes [e.g. patient deaths/readmissions]
- [ ] Identify systems errors
- [ ] Identify cognitive errors
- [ ] Teach individual professional accountability
- [ ] Discuss interesting cases
- [ ] Other:

CASE FINDING

5. How are cases for M&M identified?
Check all that apply

- [ ] Email from providers
- [ ] Hospital's patient safety reporting system
- [ ] Referred from risk management
- [ ] Regular review of deaths in the ED
- [ ] Regular review of dealths after admission
- [ ] Regular review of return visits

Other Triggers:

6. Is there a method of submitting cases anonymously for M&M?

- ( ) Yes
- ( ) No

CASE SELECTION

7. What criteria are used to decide which cases are presented?
Rank in order of importance

- [ ] Severity of Outcome
- [ ] Case involves errors, regardless of patient outcome
- [ ] Referred by another department for presentation
- [ ] Interesting nature of disease

8. Who decides which cases are presented?

- ○ Resident presenting conference
- ○ Attending supervising conference
- ○ Joint decision of resident and attending

Other (please specify)

9. Who is in charge of overseeing the M&M process?

- ○ Program director
- ○ Associate/Assistant program director
- ○ Director of Quality
- ○ Other faculty

Other (please specify)

PRESENTATION

10. Who presents the cases at M+M?

- ◯ Resident involved in patient’s care
- ◯ Resident who presents entire conference (not involved in patient’s care)
- ◯ Faculty involved in patient’s care
- ◯ Faculty not involved

Other (please specify)

* 11. Please check the frequency of the following activities or events:

	Never	Rarely	Frequently	Always
Attendings that are involved in cases are identified by name by the presenters	◯	◯	◯	◯
Residents that are involved in cases are identified by name by presenters	◯	◯	◯	◯
Clinicians that are involved in cases are present at M+M	◯	◯	◯	◯
Attendings that are involved in cases are asked to comment on the case	◯	◯	◯	◯
Residents that are involved in cases are asked to comment on the case	◯	◯	◯	◯
Analysis of systems errors contributing to each case is performed as a part of M+M conference	◯	◯	◯	◯

FOLLOW UP

12. Is there a single individual responsible for following up on systems issues identified at M+M?

- ◯ Yes
- ◯ No

13. Is there a formalized process for following up on systems issues identified at M+M?

- ◯ Yes
- ◯ No

14. Are changes that are made reported back to residents?

- ◯ Yes, at a future M&M
- ◯ Yes, by other methods (e.g. email)
- ◯ Not regularly

15. Do you believe systems issues identified in M&M conference lead to change in your emergency department?

- ◯ Always
- ◯ Sometimes
- ◯ Never

16. Is there regular debriefing for residents who have their cases discusses at M&M?

- ◯ Yes, by a member of residency administration
- ◯ Yes, by chief resident
- ◯ Yes, by someone else
- ◯ No

17. Is M&M formally evaluated by Attendings?

- ◯ Yes
- ◯ No

18. Is M&M formally evaluated by Residents?

- ◯ Yes
- ◯ No

19. Please rank the following statements

	Strongly Disagree	Disagree	Neutral	Agree	Strongly Agree
The Residents find M&M to be of educational value	○	○	○	○	○
Case discussion is focused on cognitive errors	○	○	○	○	○
Case discussion is focused on identifying systems errors	○	○	○	○	○
M&M contributes to the culture of safety at my institution	○	○	○	○	○
